# Association of the Use of a Heated Tobacco Product with Perceived Stress, Physical Activity, and Internet Use in Korean Adolescents: A 2018 National Survey

**DOI:** 10.3390/ijerph16060965

**Published:** 2019-03-18

**Authors:** Ahnna Lee, Kang-Sook Lee, Hanul Park

**Affiliations:** 1 Department of Public Health, Graduate School, The Catholic University of Korea, 222 Banpo-daero, Seocho-gu, Seoul 06591, Korea; abcde@catholic.ac.kr (A.L.); onefence@catholic.ac.kr (H.P.); 2 Department of Preventive Medicine, College of Medicine, The Catholic University of Korea, 222 Banpo-daero, Seocho-gu, Seoul 06591, Korea

**Keywords:** heated tobacco product, perceived stress, physical activity, internet use, adolescents

## Abstract

The awareness and use of the recently introduced heated product in the global tobacco market is rapidly increasing. Few studies have investigated the association of this product’s use with health-related factors. To examine the association of the heated tobacco product (HTP)’s use with perceived stress, physical activity, and internet use, we analyzed data from the Korea Youth Risk Behavior Survey using multinomial logistic regression models. The participants included 60,040 students from middle school and high school. There were significant associations between high perceived stress and cigarette use only, dual use of cigarette and e-cigarette, triple use of cigarette, e-cigarette, and HTP; a negative association between HTP’s use and perceived stress; positive association between physical activity and tobacco use; and not using the internet significantly increased the odds of use of all types of tobacco products. A smoking prevention program, tailored to meet the needs of different types of tobacco product users, is recommended. A program aimed at not only increasing awareness of perceived risk but also decreasing perceived benefits of risky behaviors, should also be considered. Further research using a longitudinal design to test the causal relationship of tobacco product use with perceived stress, physical activity, and internet use is warranted.

## 1. Introduction

Tobacco use has been a major global public health concern affecting both smokers and non-smokers [1]. Although the prevalence of cigarette smoking in Korean adolescents appears to be steadily decreasing since 2007, the rate of adolescent lifetime cigarette smoking has slightly increased from 13.7% in 2017 to 14.9% in 2018 [2]. Recently, the use of electronic cigarettes (e-cigarettes) has been one of the most important public health concerns among the youth according to reports of the US Surgeon General [3]. In Korea, there was a rapid increase of approximately twentyfold in e-cigarette use from 0.5% in 2008 to 10.1% in 2015 [4,5].

Adolescence is a transition period associated with increased risk-taking and novelty-seeking behavioral changes. Among adult daily smokers, nearly 88% first tried smoking by the age of 18 [6]. In addition, adolescence is a period characterized by the development of brain circuits that control cognition and emotion, resulting in vulnerability to nicotine and tobacco smoking [7]. Smoking initiation in younger ages is associated with increased nicotine dependence which can lead to decreased likelihood of smoking cessation [8]. Since adolescents’ brains are particularly sensitive compared to those of adults, preventing youth initiation and transition from experimenter to established smoker is critical in this period [9].

The relationship between perceived stress and tobacco use has been examined in several studies. A recent study that applied a multi-country meta-analysis revealed that perceived stress is associated with tobacco use among adults in most countries [10]. However, studies indicating the association of perceived stress and smoking in adolescents are scarce [11]. Adolescence has been characterized as a time of ‘storm and stress’ with physical, emotional, and cognitive changes. Major stressors in adolescence include family conflict, parental control, school performance, romantic relationships, peer pressure, interaction with teachers, uncertainty about the future, and financial pressure [12].

Studies on relationships between smoking and physical activity have offered conflicting results [13]. In a recent study among Canadian youth, e-cigarette use was positively associated with participating in intramural, competitive, and team sports [14]. According to a previous study conducted among young males in Finland, participating in team sports created significantly higher odds of dual use of snus and cigarettes compared to those who did not participate in team sports [15]. In contrast, several studies have revealed the negative associations between physical activity and smoking in adults [16,17]. Current smokers were significantly more likely to be physically inactive than those who never smoked according to a study conducted among adults in the US [16]. Current smoking is significantly associated with having exercised less in the past week than former or never smokers in adults over 40 years of age [17]. However, there are fewer reports related to the negative relationship between smoking and physical activity in adolescents [13].

The association between internet use and smoking has recently been studied. Both male and female adolescents have displayed a significant association between the risk of internet addiction and lifetime cigarette smoking, suggesting that internet addiction may trigger other problematic behaviors [18]. A longitudinal study on Korean adolescents revealed that excessive use of the internet at the age of 15 was significantly associated with tobacco smoking at the age of 20 [19]. However, there also has been a debate over whether spending ‘too much’ time on the internet is a sign of addiction or just a form of healthy social interaction [20]. In that perspective, what matters is the contents of internet use, not the amount of time spent online.

In spite of the changing landscape of the tobacco market, any form of tobacco product use among adolescents is never safe [3]. In 2014, one of the major tobacco companies launched a new tobacco product with a claim of a safer tobacco heating system [21]. This so-called heated tobacco product (HTP) heats a cigarette stick soaked in glycerol and propylene glycol to up to 350 °C, producing aerosol. Despite the “less harmful” claim by the tobacco company, the aerosol from the heated tobacco product still contains numerous harmful constituents such as acrolein, acetaldehyde, and formaldehyde [22]. The levels of nicotine in this HTP cigarette stick are similar to those in combustible cigarettes [23]. Furthermore, a HTP cigarette stick also includes a flavored agent which is commonly detected in e-cigarettes that appeal to adolescents for experimentation [24]. In a recent research on flavoring chemicals in e-cigarettes, diacetyl which has been associated with the development of bronchiolitis obliterans was detected above the laboratory limit in 39 out of the 51 evaluated flavors [25]. Nevertheless, HTP is often marketed as a low risk and familiar personal electronic device, not as a harmful substance which appeals to the youth [26].

Since HTP was introduced in Korea in April 2017, there is no published study on its use using national statistics. Moreover, research on tobacco products have concentrated more on conventional cigarettes since HTP products are relatively new to the tobacco market. Several laboratory studies or randomized control trials have been conducted on HTP; however, no study has examined the association of the use of HTP with perceived stress, physical activity, and internet use. Therefore, considering the importance of teenage years and increasing worldwide popularity of HTP, examining this association among adolescents, using nationwide population-based studies, is necessary. Using the Korean national representative data, the main objectives of this study are as follows: (1) to assess the current status of the use of tobacco products, including HTP, in Korean adolescents; and (2) to examine whether the use of HTP is associated with perceived stress, physical activity, and internet use.

## 2. Materials and Methods

### 2.1. Participants

This study used data from the 14th (2018) Korea Youth Risk Behavior Survey (KYRBS) conducted by the Korea Centers for Disease Control and Prevention (KCDC). The survey is a government approved statistical survey that has been performed annually since 2005 to monitor health behaviors such as tobacco smoking, alcohol drinking, oral health, obesity, and physical activity in adolescents. The survey utilized a stratified multi-stage cluster sampling design to produce a national representative sample of Korean adolescents. Of the total 2,850,118 students from 5625 middle and high schools in Korea, a countrywide representative sample of 62,823 youth aged 12–18 years was selected. In the primary unit, 400 middle and 400 high schools were selected from 117 strata that were derived using administrative regions and school characteristics. Subsequently, one class in each school grade was randomly selected from each sampled school for the secondary unit.

Students were first asked about their willingness to participate and only those who agreed to participate proceeded with the survey. Of the 62,823 participants, 60,060 students (aged 12 to 18) from a total of 800 schools (400 middle school and 400 high school) participated in the survey with a response rate of 95.6%. Participants had the right to withdraw from the survey at any time with no penalties. As teachers fully explained the process and purpose of the survey, students completed the web-based anonymous self-administered survey. Students with low literacy levels, dropouts, and extended absences were excluded. Participants did not fill-in any personal information such as names, phone numbers, addresses, or social security numbers. Students checked informed consent online before taking part in the survey. Parental consent was exempted because the survey was conducted in schools with large numbers of participants. This study was approved by the Institutional Review Board of the Catholic University of Korea (MC18ZESI0112).

### 2.2. Measures

#### 2.2.1. Socio-Demographic Variables

The general characteristics of participants were gender, school year, residential area, perceived academic achievement, perceived family economic status, and lifetime alcohol drinking. Participants reported their gender as either female or male. Responses for the school year were classified from “middle first, middle second, middle third, high first, high second, and high third “to “middle school and high school”. Responses regarding residential area were divided into three categories based on city types: Large cities, medium or small cities, and provinces. Perceived academic performance and perceived family economic status were initially assessed as “high, mid-high, middle, mid-low, and low” and re-categorized as “high, middle, and low”. Students were asked to answer whether they had ever attempted to drink alcohol, even once, in their lifetime. Response options were “yes” and “no”.

#### 2.2.2. Tobacco Ever-Use Categories

In this study, we assessed the experience of the use of tobacco products during the participants’ lifetime to evaluate the smoking patterns of adolescents. Lifetime cigarette use was examined using the following question: “Have you ever tried even one or two puffs of a cigarette in your life?” Participants who responded “yes” to the question were defined as a “cigarette ever-user”. Lifetime e-cigarette use was assessed with the following question: “Have you ever used e-cigarettes in your life?” Students who responded “yes” to the question were classified as an “e-cigarette ever-user”. Lifetime HTP use was evaluated with the following question: “Have you ever used heated tobacco products in your life, excluding conventional cigarettes and e-cigarettes?” Individuals who answered, “yes” to this question were regarded as a “HTP ever-user”. A single use of a tobacco product was defined as reporting the use of only one among the three tobacco products. Dual use refers to the use of two forms of tobacco products. Reporting ever-use of all the three products (cigarette, e-cigarette, and HTP) was defined as triple use.

#### 2.2.3. Perceived Stress

Perceived stress was evaluated using the following question: “How would you rate your usual stress level?” The response options were “very high”, “high”, “moderate”, “low”, and “none”, and regrouped into three categories: “very high/high” as “high”, “moderate”, and “low/none” as “low”. 

#### 2.2.4. Physical Activity

Physical activity was assessed using the following question: “On how many of the past seven days did you participate in vigorous-intensity physical activity that made you sweat and breathe hard, such as soccer, basketball, jogging, bicycling, swimming, and Taekwondo, for at least 20 min?” The frequency of physical activity ranged from “no participation” to “more than five days per week”. Participating in vigorous-intensity physical activity for at least three days per week was classified as physically active, based on the moderate level physical activity criteria from the International Physical Activity Questionnaire (IPAQ) [27].

#### 2.2.5. Internet Use

Internet use was assessed through the following question: “In the past 30 days, have you used the internet for non-educational purposes?” Questions were asked for both weekdays and the weekend, and the participants were to answer them, respectively. Respondents were categorized into two based on their internet use status. Any use reported on either a weekday or the weekend was classified as “yes”. No use reported both on weekdays and the weekend were classified as “no”.

### 2.3. Statistical Analysis

All statistical analyses in this study were performed using SPSS version 22.0 (IBM, Armonk, NY, USA), which helped to reflect complex sampling modules, considering the stratification variables, cluster variables, and weight. First, descriptive statistics of the adolescents’ general characteristics were presented as unweighted frequency and weighted percentages. Subsequently, chi-square tests were performed to examine the differences among the tobacco ever-use categories according to the general characteristics, perceived stress, physical activity, and internet use. Lastly, a multinomial logistic regression analysis, adjusted for gender, grade, residential area, perceived academic achievement, perceived family economic status, and alcohol drinking was conducted to investigate the associations of the use of HTP with perceived stress, physical activity, and internet use. For this study, statistical significance was set at *p* < 0.05.

## 3. Results

### 3.1. General Characteristics

Table 1 displays the general characteristics of participants according to tobacco ever-use categories. Overall, males represent higher prevalence smoking rates in almost all types of tobacco products than females. More high school students experienced the use of tobacco products than middle school students. Students living in large cities indicated lower rates of smoking cigarettes only or e-cigarettes only than those living in medium or small cities and provinces. Students with low academic achievements had significantly higher tobacco ever-use experiences than those with high achievements. The prevalence rates of cigarette only and dual use of cigarettes and e-cigarettes were higher among students with low perceived family economic status as compared to those with high and middle perceived family economic status.

### 3.2. Perceived Stress, Physical Activity, and Internet Use According to Tobacco Ever-Use Categories

The prevalence rate for perceived stress, physical activity, and internet use by tobacco ever-use categories is indicated in Table 2. Students with high perceived stress displayed higher prevalence rates of smoking cigarettes only, dual use of cigarettes and e-cigarettes, and triple use of cigarettes, e-cigarettes, and HTPs than those with moderate or low perceived stress. HTP only users were more likely to report low perceived stress than moderate or high perceived stress. In terms of physical activity, subjects performing vigorous physical activity more than three times per week displayed a higher prevalence rate of tobacco use in all types of tobacco products than the group performing vigorous physical activity less than three times per week. Regarding internet use, students reporting no use of the internet both on weekdays and the weekend displayed higher prevalence rates of use of all types of tobacco products compared with the group using the internet either on weekdays or the weekend.

### 3.3. Association of Tobacco Use with Mental Health and Internet Use

Table 3 presents the association of tobacco ever-use with perceived stress, physical activity, and internet use. Students with high perceived stress had higher odds of using cigarettes only, dual use of cigarettes and e-cigarettes, and triple use of cigarettes, e-cigarettes, and HTPs (odds ratio [OR] = 1.11, 1.17, and 1.34). Moderate perceived stress was negatively associated with the use of HTP only compared to low perceived stress (OR = 0.47, 95% CI: 0.24–0.93). In terms of physical activity, positive associations were presented in general. Students reporting participation in vigorous-intensity physical activities more than or equal to three times per week were more likely to use all types of tobacco products except for cigarette only. Not using the internet on both weekdays and the weekend displayed a significantly positive association with the use of all types of tobacco products.

## 4. Discussion

Despite the fact that HTP is not widespread compared to conventional cigarettes or e-cigarettes, its awareness and use has been notably increasing [28]. Our results demonstrate that the prevalence of lifetime HTP use was 2.9%, including single use (0.1%), dual use (0.5%), and triple use (2.3%). When compared to a previous study conducted in Japan, 6.6% of the adults were HTP ever-users and lifetime HTP use was lower among Korean adolescents [29]. In the US, 2.2% of adults have reported HTP ever-use, despite HTP not yet being commercially introduced [28]. However, no studies, so far, have investigated lifetime HTP use including dual or poly use in adolescents. To the best of our knowledge, this is the first study to analyze the prevalence of tobacco product ever-use, including HTP, among Korean adolescents.

In the current study, high levels of perceived stress indicate an increased likelihood of being a tobacco product user of conventional cigarettes, dual use of cigarette and e-cigarette, and triple use of cigarette, e-cigarette, and HTP. The positive association between perceived stress and cigarette smoking found in this study is consistent with previous studies [10,30]. According to several theories, perceived stress is often associated with increased hypothalamus–pituitary–adrenal axis reactivity and craving for nicotine [31]. Smoking is also often used as a stress-relieving strategy in daily life [32]. Adolescents in Korea especially experience extreme academic stress with excessive competition and spend a great deal of time studying for high academic achievement in preparation for college entrance [33,34].

However, in this study, subjects who used HTP only were less likely to report perceived stress, suggesting experimental use of tobacco products. HTP, relatively new in the tobacco market, and electronic products promoted with a novel and high-technology image may appeal to the youth [26]. Reasons for adolescents to start smoking include curiosity, social norms, offers or pressures, enhancement of self-image, reduction of negative affect, boredom, and habit. Curiosity was the most commonly reported reason for initiating smoking by 45% of adolescents [35]. A growing number of adolescents in the US are experimenting with e-cigarettes, even with no experience of conventional cigarettes [36,37]. Highlighting the difference with conventional cigarettes, HTP is marketed as a “harm-reduction” and “smokeless” tobacco product [20]. With limited literature regarding the health effects of HTP, consumers may be misled by the reduced health-risk advertising [26]. A previous study conducted in the United Kingdom revealed that the never-smokers who have tried e-cigarettes are associated with an elevated risk of onset of conventional cigarette smoking compared with the never-smokers who have not tried e-cigarettes [38]. Careful monitoring is necessary for youth experimentation with novel nicotine products, including HTP.

Our study revealed that physically active adolescents were more likely to smoke. Our results indicate an inverse association in comparison to approximately 60% of previous studies that revealed a negative association [13]. There have been few studies providing explanations for why positive associations between physical activity and tobacco use have been found [39,40]. Two reasons to explain why certain behaviors that seemed to be contradictory could coexist were suggested. (1) Health-conscious smokers may adopt physical activity as a harm-reduction method. (2) Both physical activity and smoking were used as a weight-control strategy [40]. Decision-making on risk behavior is based on a balance of both risk and benefit perception. Prevention programs should not only aim to raise the awareness of perceived risk but also decrease perceived benefits of risky behaviors [41].

Another possible reason for the positive association is that engaging in physical activities may provide increased opportunities for group gathering that can lead to smoking initiation [42]. Burke et al. offered an explanation on young Australian men, members of a sports club, participating in social activities where binge drinking and tobacco use commonly occur, suggesting a positive association between physical activity and smoking [39]. Peer influence in adolescents is one of the top reasons given for why an individual would pick up smoking in the context of peer groups [43]. Recent studies established that many adolescents not only owned a vaping product but also borrow the same from other people, making it part of a social experience [44].

Another important finding in this study is that not using the internet on both weekdays and the weekend was positively associated with all types of use of tobacco products. Internet addiction or problematic internet use is referred to as a loss of control in using the internet, which can result in psychosocial problems [45]. Internet addiction or problematic internet use is often considered as a predictor of substance use, such as tobacco use [46,47]. However, in comparison to excessive internet gaming, frequent but non-pathological internet use can lead to better digital competence among youth [48]. Information and communication technologies causing major and minor changes in society have become inevitable phenomena in adolescents’ daily lives [49].

Non-academic purpose of using the internet in adolescents include socialization, psychological needs, and seeking entertainment [50]. With diverse intentions for using the internet, time spent online itself may not imply behavioral addiction or an issue of concern. The internet serves as a complex communication tool and provides the possibility of building intimate social environments. A previous study found that socially rich individuals with strong ties utilize online communication more frequently, and as a result, they maintain or build stronger solidarity than socially isolated adolescents [51]. The benefits of using the internet, such as social connectedness or elevated self-esteem in adolescents, may prevent them from involving themselves in unhealthy behavior.

### Limitations and Strengths

In this study, there were several limitations. First, the current study used a cross-sectional research design which could not confirm causal relationships between the use of tobacco products and perception of stress, physical activity, and the use of the internet. Secondly, the results of this study could be somewhat biased because the survey was in a self-reported format. Respondents tend to under-report socially undesirable behaviors, such as tobacco use, especially the female students, even though the survey was conducted anonymously. Lastly, the criteria for vigorous-intensity physical activity may be relative depending on physical conditions, such as having asthma or other respiratory diseases. Despite these listed limitations, this is the first study analyzing the association of the use of a heated tobacco product with perceived stress, physical activity, and internet use among Korean adolescents using national representative statistics, with a high response rate of over 95%. With the administrative support of the Ministry of Education, it was possible to achieve a high response and low refusal rate [52]. Also, the web-based survey was designed to be proceeded and completed only when each question was answered, suggesting a low probability of missing data. The results from the survey participants can be generalized to the total adolescent population.

## 5. Conclusions

Despite several limitations, our study contributes to the existing relevant literature by investigating the use of tobacco products, including HTP, in relation to perceived stress, physical activity, and internet use. The results indicate that adolescents with high perceived stress, high frequency of physical activity, and who spend less time using the internet were more likely to engage in the use of tobacco products. Further research, using a longitudinal design, is needed to determine whether this relationship is temporal. Considering the growing population of ever-using HTP, further investigation of long-term health effects of HTP should be performed to provide information to health policy makers and parents. A smoking prevention program designed to meet the needs of different types of tobacco products users should be considered. A program aimed to not only raise the awareness of perceived risk but also decrease perceived benefits of risky behaviors is recommended.

## Figures and Tables

**Table 1:** General characteristics according to tobacco ever-use categories.

Variable	Category	Tobacco Ever-Use Categories								*p*
		*N* (Weighted %)								
		Never-Smoke	Cigarette Only	E-Cigarette Only	HTP Only	Cigarette + E-Cigarette	Cigarette + HTP	E-Cigarette + HTP	Cigarette + E-Cigarette + HTP	
Total		50,778 (83.8)	4690 (7.9)	571 (1.0)	59 (0.1)	2433 (4.4)	147 (0.3)	92 (0.2)	1270 (2.3)	
Gender	Male	23,770 (77.0)	3074 (10.2)	434 (1.5)	41 (0.1)	1951 (6.9)	100 (0.4)	72 (0.3)	1021 (3.7)	<0.001
	Female	27,008 (91.3)	1616 (5.4)	137 (0.4)	18 (0.1)	482 (1.7)	47 (0.2)	20 (0.1)	249 (0.9)	
Grade	Middle	27,426 (90.5)	1681 (5.6)	197 (0.6)	23 (0.1)	643 (2.3)	31 (0.1)	24 (0.1)	204 (0.7)	<0.001
	High	23,352 (78.1)	3009 (9.9)	374 (1.3)	36 (0.1)	1790 (6.2)	116 (0.2)	68 (0.2)	1066 (3.7)	
Residential area	Large city	22,667 (84.6)	1995 (7.6)	230 (0.8)	22 (0.1)	1014 (4.0)	60 (0.2)	52 (0.2)	614 (2.4)	0.009
	Medium, small city	24,333 (83.5)	2290 (8.0)	286 (1.1)	32 (0.1)	1253 (4.8)	75 (0.3)	26 (0.1)	555 (2.2)	
	Province	3778 (81.4)	405 (9.4)	55 (1.4)	5 (0.1)	166 (4.3)	12 (0.3)	4 (0.1)	101 (3.0)	
Academic performance	High	20,772 (88.2)	1386 (5.9)	185 (0.8)	32 (0.2)	634 (3.0)	49 (0.2)	36 (0.2)	326 (1.5)	<0.001
	Middle	15,195 (85.8)	1278 (7.6)	148 (0.9)	14 (0.1)	584 (3.7)	31 (0.2)	21 (0.1)	255 (1.7)	
	Low	14,811 (76.8)	2026 (10.6)	238 (1.3)	13 (0.1)	1215 (6.8)	67 (0.4)	35 (0.2)	689 (4.0)	
Family economic status	High	20,720 (84.9)	1699 (7.1)	238 (1.0)	32 (0.1)	893 (4.0)	59 (0.3)	41 (0.2)	525 (2.4)	<0.001
	Middle	23,799 (84.7)	2123 (7.9)	237 (0.9)	16 (0.1)	1055 (4.2)	60 (0.2)	34 (0.2)	484 (1.9)	
	Low	6259 (77.4)	868 (10.6)	96 (1.3)	11 (0.1)	485 (6.4)	28 (0.4)	17 (0.2)	261 (3.6)	
Alcohol drinking	Yes	17,033 (68.2)	3597 (14.4)	404 (1.7)	34 (0.1)	2211 (9.4)	132 (0.5)	68 (0.3)	1218 (5.3)	<0.001
	No	33,745 (95.3)	1093 (3.1)	167 (0.5)	25 (0.1)	222 (0.7)	15 (0.0)	24 (0.1)	52 (0.2)	

**Table 2:** Perceived stress, physical activity, and internet use according to tobacco ever-use categories.

Variable	Category	Tobacco Ever-Use Categories								*p*
		*N* (Weighted %)								
		Never-Smoke	Cigarette Only	E-Cigarette Only	HTP Only	Cigarette + E-Cigarette	Cigarette + HTP	E-Cigarette + HTP	Cigarette + E-Cigarette + HTP	
Total		50,778 (83.8)	4690 (7.9)	571 (1.0)	59 (0.1)	2433 (4.4)	147 (0.3)	92 (0.2)	1270 (2.3)	
Perceived stress	High	20,188 (82.5)	2087 (8.6)	238 (1.0)	24 (0.1)	1071 (4.7)	66 (0.3)	35 (0.1)	603 (2.7)	<0.001
	Moderate	21,273 (85.1)	1762 (7.3)	219 (0.9)	16 (0.1)	931 (4.1)	54 (0.2)	39 (0.2)	444 (2.0)	
	Low	9417 (83.9)	841 (7.7)	114 (1.1)	19 (0.2)	431 (4.4)	27 (0.3)	18 (0.2)	233 (2.2)	
Physical activity	≥3 times/week	31,886 (85.9)	2663 (7.4)	280 (0.8)	22 (0.1)	1261 (3.7)	77 (0.2)	42 (0.1)	629 (1.9)	<0.001
	<3 times/week	18,892 (80.4)	2027 (8.8)	291 (1.3)	37 (0.2)	1172 (5.5)	70 (0.3)	50 (0.3)	641 (3.1)	
Internet use	No	8215 (75.2)	1159 (10.7)	163 (1.6)	22 (0.2)	672 (6.7)	50 (0.5)	39 (0.4)	454 (4.8)	<0.001
	Yes	42,563 (85.7)	3531 (7.3)	408 (0.9)	37 (0.1)	1761 (3.9)	97 (0.2)	53 (0.1)	816 (1.8)	

**Table 3:** Multinomial logistic regression examining perceived stress, physical activity, and internet use by tobacco ever-use patterns.

Tobacco Ever-Use Categories		Cigarette Only	E-Cigarette Only	HTP Only	Cigarette + E-Cigarette	Cigarette + HTP	E-Cigarette + HTP	Cigarette + E-Cigarette + HTP
Variable	Category							
Perceived stress	High	1.11 [1.02, 1.22]	1.05 [0.82, 1.35]	0.79 [0.41, 1.52]	1.17 [1.03, 1.33]	0.93 [0.60, 1.45]	0.74 [0.38, 1.46]	1.34 [1.13, 1.58]
	Moderate	0.92 [0.84, 1.00]	0.87 [0.70, 1.10]	0.47 [0.24, 0.93]	0.93 [0.82, 1.05]	0.76 [0.48, 1.21]	0.83 [0.44, 1.56]	0.90 [0.76, 1.05]
	Low	1	1	1	1	1	1	1
Physical activity	≥3 times/week	1.06 [0.99, 1.13]	1.33 [1.11, 1.60]	3.68 [1.68, 5.60]	1.15 [1.05, 1.25]	1.52 [1.08, 2.14]	1.66 [1.09, 2.51]	1.36 [1.19, 1.55]
	<3 times/week	1	1	1	1	1	1	1
Internet use	No	1.55 [1.43, 1.67]	1.90 [1.59, 2.26]	2.95 [1.76, 4.97]	1.77 [1.59, 1.97]	2.43 [1.78, 3.34]	3.41 [2.34, 4.97]	2.66 [2.35, 3.01]
	Yes	1	1	1	1	1	1	1

1 Note: Never-smoker as a reference category. Adjusted for gender, grade, residential area, academic performance, family economic status, and alcohol drinking.
